# Does upper limb dominance influence outcomes in adults undergoing primary anatomic or reverse total shoulder arthroplasty? A systematic review

**DOI:** 10.1016/j.jsea.2026.100023

**Published:** 2026-04-21

**Authors:** Periklis Giannakis, Mitchell A. Johnson, Sara E. Covin, Olivia M. Jochl, Sophia T. Zhuang, Jashvant Poeran, Robert G. Marx, Samuel A. Taylor, Lawrence V. Gulotta

**Affiliations:** a Department of Anesthesiology, Critical Care and Pain Management, Hospital for Special Surgery, New York, NY, USA; b Sports Medicine Institute, Hospital for Special Surgery, New York, NY, USA; c School of Medicine, University of North Carolina, Chapel Hill, NC, USA; d School of Medicine, Weill Cornell University, New York, NY, USA

**Keywords:** Total shoulder arthroplasty, Reverse shoulder arthroplasty, Limb dominance, Handedness, Range of motion, Patient-reported outcomes, Radiographic outcomes, Acromial stress fracture

## Abstract

**Background:**

While several patient-specific factors have been shown to influence anatomic and reverse total shoulder arthroplasty (aTSA and rTSA) outcomes, the effect of operated limb dominance remains unclear. Therefore, we performed a systematic review to examine the effect of upper limb dominance on post-operative outcomes.

**Methods:**

We queried PubMed, Embase, and Cochrane to identify relevant level IV or greater studies on adults. Risk-of-bias was assessed with Risk Of Bias In Non-Randomized Studies of Exposure. Results were qualitatively synthesized by outcome.

**Results:**

Thirty studies (7,916 shoulders, 7,897 patients) were included. Two out of 4 studies demonstrated greater forward elevation and internal rotation on the dominant arm after aTSA. No association between arm dominance and post-operative patient-reported outcome measures was identified in 14 studies. Adverse rTSA outcomes on the dominant side were more likely to be associated with greater dissatisfaction and loss of independence in 3 studies. One out of 3 studies showed greater incidence of radiolucent lines in dominant-side implants at 10-year follow-up. Some studies indicated a higher risk of acromial stress fractures for dominant limb rTSAs, while studies had conflicting results regarding subacromial notching. There was no difference in revision rates in 4 studies.

**Conclusion:**

In this systematic review, dominant arm surgery, particularly after aTSA, may facilitate earlier functional recovery but could also be associated with long-term radiographic changes. Nevertheless, arm dominance does not appear to influence patient-perceived clinically meaningful improvement. A potential association with early acromial stress fracture after rTSA was also observed. These findings may inform patient counseling and serve as a basis for future research.

Demand for anatomic and reverse total shoulder arthroplasty (aTSA and rTSA) has increased over the past decade due to their efficacy in treatment of advanced glenohumeral osteoarthritis, rotator cuff tear arthropathy, irreparable rotator cuff tears, and proximal humeral fractures.^6^^,^^21^ While shoulder arthroplasty is associated with good outcomes, certain patient-specific factors, such as age, sex, previous shoulder surgery, surgical indication, diabetes and smoking, have all been shown to influence outcomes after TSA.^1^^,^^2^^,^^8^^,^^16^^,^^23^^,^^24^^,^^34^

A relatively understudied factor is the laterality of the surgical site compared to the dominant limb. Intuitively, shoulder surgery on the dominant limb should result in different outcomes due to differences in use, needs, dexterity, and importance for activities of daily living. This has been confirmed for arthroscopic management of shoulder conditions such as anterior glenohumeral instability, rotator cuff disorders, and throwing-athlete disorders, with studies demonstrating that upper limb dominance primarily influences range of motion (ROM) and patient-reported disability.^4^^,^^54^^,^^68^

However, few studies have examined whether limb dominance affects outcomes following TSA, with often contradictory reports regarding functionality,^12^^,^^17^ radiographic outcomes,^13^ post-operative ROM, strength, and patient-reported outcome measures (PROMs).^5^^,^^26^ These conflicting results may reflect small sample sizes, heterogeneous populations, and differing methodologies, which underscores the need for a larger, systematic evaluation of the existing literature to better understand the expected post-operative courses for these patients.

Therefore, we aimed to evaluate the effect of operated limb dominance on post-operative (1) symptoms and physical examination, (2) PROMs, (3) radiographic outcomes, (4) satisfaction and activities of daily living, and (5) complications in adults undergoing primary aTSA or rTSA.

## Materials and methods

### Protocol registration and eligibility criteria

This systematic review was prospectively registered in PROSPERO (CRD420251012188) and adhered to the Preferred Reporting Items for Systematic reviews and Meta-Analyses 2020 checklist.^45^ The full protocol and registration are available in the Supplementary Material.

Studies were included if they were written in or translated to English and were original studies of level IV evidence and above. Case reports, systematic reviews, expert opinions, and studies classified as Level V evidence were excluded. In addition, studies with irretrievable full texts and abstracts or meeting proceedings without full-text availability were also excluded.

All studies including adults undergoing primary aTSA or rTSA for any surgical indication were assessed for eligibility. Studies were included if (1) dominant vs. nondominant limb TSA was the primary comparison of interest, or (2) limb dominance was analyzed as an additional variable with extractable data. Studies with a minimum of 1-year follow-up were eligible for inclusion. A prespecified list of eligible outcomes corresponding to our study aims was used to determine inclusion or exclusion of studies.

### Information sources and search strategy

The last systematic search was conducted on March 15, 2026. Bibliographic databases searched were PubMed, Embase, and the Cochrane library. Backward citation searching was performed for all studies included in the data extraction phase to help ensure comprehensive study inclusion and completeness of the information presented. The finalized search strategy combined Medical Subject Headings terms and free-text keywords using Boolean operators. The PubMed search strategy was manually developed and then adapted for other searched databases by two reviewers (PG and OMJ). The full search strategies can be found in Supplementary Material.

### Selection process

After removing duplicates, all eligible manuscripts were imported into the Cochrane-approved software Covidence (Veritas Health Innovation, Deerfield, IL). Title and abstract screening and full-text review were conducted independently by two reviewers (OMJ and STZ). Any discrepancies were discussed and resolved among the two reviewers; unresolved disagreements were settled by a third independent reviewer (PG).

### Data collection

Two independent reviewers (PG and SEC or OMJ) collected data from each report using a prespecified template; any discrepancies were first discussed between the two reviewers, and in case of withstanding disagreement, a third independent reviewer (MAJ) resolved conflicts.

Extracted data included study identification variables, method details, population details, and baseline characteristics (full list of prespecified variables in Supplementary Material.) Physical examination findings included ROM and strength. The prespecified list of PROMs included the American Shoulder and Elbow Surgeons Score (ASES),^39^ self-reported pain in the numeric rating scale or visual analog scale (VAS),^44^ the Constant Murley Score (CMS),^14^ the Simple Shoulder Test (SST),^56^ the Shoulder Pain and Disability Index (SPADI),^7^ the Disabilities of the Arm, Shoulder and Hand (DASH) or quickDASH,^15^ and the University of California and Los Angeles Shoulder Score.^30^ Radiographic outcomes, including the presence of radiolucent lines (RLLs) and component wear, were recorded. In addition, post-operative complications such as acromial stress fractures (ASFs) and scapular notching were classified as complications. RLLs were not considered complications as their symptoms and clinical impact is debatable.^3^^,^^25^^,^^59^ Rate of revision arthroplasty was also recorded. Self-reported outcomes regarding satisfaction, activities of daily living, and independence were extracted.

### Study risk of bias assessment

The Risk Of Bias In Non-Randomized Studies-of Exposures version 2^60^ was used. The tool includes a preliminary step to determine whether a full assessment is required, as certain features (eg, confounding or outcome measurement) may directly classify a result as high risk-of-bias. When fully performed, Risk Of Bias In Non-Randomized Studies-of Exposures evaluates 7 domains: confounding, exposure classification, selection bias, deviations from intended interventions, missing data, outcome measurement, and selective reporting. Domain-specific and overall risk-of-bias was characterized as “low,” “some concerns” and “high.” A prespecified list of confounding factors included patient-specific factors (younger age, osteoporosis, obesity, smoking, male sex, previous shoulder surgery, comorbidities), surgical indication, and surgical variables (glenoid retroversion, larger humeral stem size, larger glenosphere diameter, uncemented humeral fixation).^1^^,^^2^^,^^8^^,^^16^^,^^23^^,^^24^^,^^34^^,^^49^^,^^58^^,^^66^ Two independent assessors (PG and SEC or OMJ) assessed risk-of-bias and conflicts were solved by consensus or by a third independent assessor (MAJ). Risk-of-bias was then visualized using the robvis tool.^38^

### Synthesis methods

Nonoutcome study characteristics were summarized descriptively. Outcome data was grouped by study aim and compared. Summary estimates and precision metrics were reported as dominant vs. nondominant, unless specified otherwise. Given the heterogeneity in study design, surgical indications, procedures, and study periods among the included studies, as well as the risk-of-bias assessment, a meta-analysis was not performed.

## Results

### Study selection

**Figure 1:**
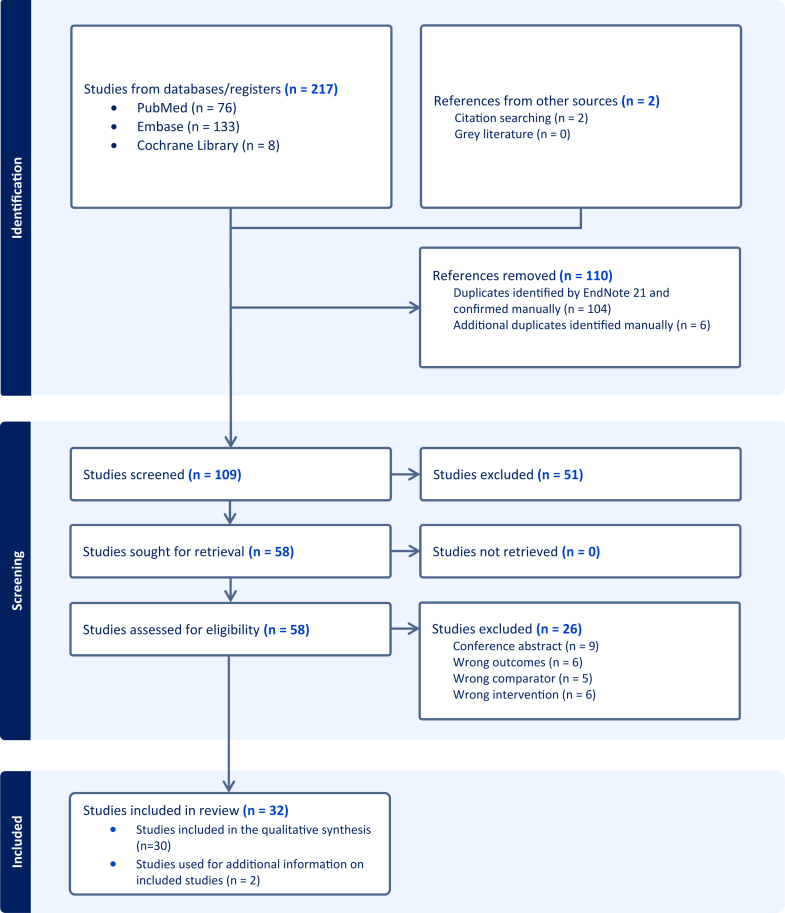
PRISMA flowchart. *PRISMA*, Preferred Reporting Items for Systematic reviews and Meta-Analyses.

A total of 219 records were retrieved; 109 titles and abstracts were screened; 58 full texts were assessed; and 32 studies were included in the review. A total of 30 studies were included in the qualitative synthesis,^5^^,^^9^, ^10^, ^11^, ^12^, ^13^^,^^17^, ^18^, ^19^^,^^22^^,^^26^, ^27^, ^28^, ^29^^,^^32^^,^^33^^,^^35^, ^36^, ^37^^,^^40^^,^^42^^,^^43^^,^^46^^,^^47^^,^^50^^,^^51^^,^^57^^,^^63^^,^^67^ and two studies were used to provide additional information for data extraction (Fig. 1).^31^^,^^64^

### Study characteristics

A total of 7,916 shoulders (7,897 patients) were included, with 2,148 aTSAs and 5,768 rTSAs. Most of the studies were conducted in the United States (n = 13, 43.3%) followed by France (n = 10, 33.3%), the rest of Central Europe, Canada, and the Republic of Korea (Table I). Five studies were prospective,^12^^,^^13^^,^^22^^,^^26^^,^^50^ and sample sizes ranged from 16^36^ to 2,152^33^ patients. Twelve studies focused on aTSAs,^5^^,^^13^^,^^17^^,^^18^^,^^26^^,^^32^^,^^35^^,^^50^, ^51^, ^52^^,^^57^^,^^67^ 15^9^, ^10^, ^11^, ^12^^,^^19^^,^^27^, ^28^, ^29^^,^^36^^,^^37^^,^^40^^,^^42^^,^^43^^,^^46^^,^^47^ on rTSAs and 3^22^^,^^33^^,^^63^ on both (Table II). Mean age ranged from 57.9 ± 5.3 years^67^ to 75.2 ± 7.7 years,^19^ and follow-up ranged from 12 months^22^^,^^33^^,^^46^^,^^47^^,^^52^ to 15 years^50^ (Table III).

**Table I:** Study characteristics.

Study	Sponsorship source	Country	Setting	Start date	End date
Berthold et al 2020^5^	Arthrex	United States of America	Academic	Jun-08	Aug-12
Cazeneuve et al 2011^9^	No funding	France	Tertiary center	Jan-93	Dec-09
Cazeneuve et al 2012^10^	No information	France	Tertiary center (public)	Jan-93	May-10
Cho et al 2021^11^	National Research Foundation of Korea	Korea (Republic of)	Multicenter	Mar-09	May-18
Collin et al 2017^12^	No funding	France	Tertiary center	Jan-11	Jan-12
Collin et al 2011^13^	No funding	France	Tertiary center	1997	1999
Cvetanovich et al 2015^17^	Arthrex, Smith and Nephew, and Donjoy.	United States of America	Academic	Jan-08	Sep-11
Dauzère et al 2017^18^	Wrigth-Tomier Inc	France	Academic	Jun-12	Jan-14
DeVito et al 2019^19^	No funding	United States of America	Academic	2007	2015
Flurin et al 2024^22^	No funding	France	Sports medicine clinic	Jan-19	Apr-21
Hetto et al 2025^26^	No funding	Germany	Tertiary center	Nov-02	Mar-09
Huber et al 2020^27^	No funding	Switzerland; Germany; France	Multicenter		
Katz et al 2016^28^	No funding	France	Multicenter	Oct-03	Oct-12
Kim et al 2024^29^	No funding	United States of America	Academic	Jan-21	Jun-22
Lapner et al 2015^31^	Tornier Company	Canada	Multicenter	Nov-06	Jun-09
Lazaridou et al 2026^33^	No funding	Switzerland	Tertiary center	Jan-06	Apr-24
Mahony et al 2018^35^	No funding	United States of America	Academic	2007	2013
Mattiassich et al 2013^36^	No funding	Austria	Academic	Jan-08	Dec-11
McFarland et al 2021^37^	No funding	United States of America	Academic	Jan-09	Dec-16
Mollon et al 2017^40^	Exactech, Inc.	United States of America	Academic	2007	2014
Noble et al 2025a^42^	No funding	United States of America	tertiary care center	Dec-11	Dec-22
Noble et al 2025b^43^	Arthrex	United States of America	tertiary care center	2016	2021
Pak et al 2024a^46^	Arthrex	United States of America	Multicenter database	2015	2021
Pak et al 2024b^47^	Arthrex	United States of America	Multicenter database	2015	2021
Raiss et al 2014^50^	Stiftung Endoprothetik	France	Academic	Sep-91	Feb-95
Raiss et al 2018^51^	No funding	France	Academic	Jun-12	Dec-14
Razmjou et al 2018^52^	No funding	Canada	Academic		
Schnetzke et al 2019^57^	No funding	Germany	Academic	Aug-12	Jun-14
Vegas et al 2023^63^	No funding	United States of America	Tertiary center	Jan-09	Jan-19
White et al 2026^67^	No funding	United States of America	Tertiary center	Feb-16	Dec-21

**Table II:** Study methods.

Study	Procedure	Surgical indication	Other eligibility criteria	Total sample size	Number of withdrawals	Reason for withdrawals	Loss to follow-up	Included in analysis	Design	Unit of allocation	Minimum follow-up
Berthold et al 2020^5^	aTSA	Osteoarthritis with intact rotator cuff	Inclusion intact rotator, cuff healthy enough to undergo the procedure, willing to follow post-operative restrictions and supervised physical therapy regimen. Exclusion prior TSA, prior hemiarthroplasty, prior humeral or glenoid resurfacing	44 (40)	0	NA	2	42	Retrospective cohort study	Shoulder	12 mo
Cazeneuve et al 2011^9^	rTSA	Osteoporotic displaced comminuted fracture of the proximal humerus	NR	49	12	Deceased	2	35	Retrospective case-series	Individual	12 mo
Cazeneuve et al 2012^10^	rTSA	Isolated displaced three- or four-part fractures or fracture dislocations according to the Neer classification and the inability to obtain efficient fixation of the tubercles due to comminution and osteoporosis	NR	52	12	Deceased	3	37	Retrospective case-series	Individual	12 mo
Cho et al 2021^11^	rTSA	Any indication	NR	1,195	0	NA	408	787	Case-control study	Individual	12 mo
Collin et al 2017^12^	rTSA	Cuff tear arthropathy, irreparable massive cuff tear, binconcaveglenoid with static posterior dislocation	*Exclusion* incomplete documentation, any pre-operative diagnoses that are known to limit post-operative ROM, a transdeltoid approach, pre-operative infection.	104	3	2 declined to participate, 1 deceased	0	101	Prospective cohort study	Individual	24 mo
Collin et al 2011^13^	aTSA	Osteoarthritis with intact rotator cuff	Exclusion history of trauma (fracture or soft-tissue injury), instability (treated surgically or nonsurgically), or prior shoulder surgery were excluded. Also excluded were any shoulders with marked rotator cuff pathology defined by the presence of acromiohumeral arthritis or a full thickness rotator cuff tear and those with glenoid bone erosion where reaming and glenoid preparation did not achieve implant stability and required bone grafting at the time of arthroplasty.	66	5	Deceased	4	56 (54)	Prospective cohort study	Shoulder	10 y
Cvetanovich et al 2015^17^	aTSA	Osteoarthritis	NR	149	0	NA	29	120	Retrospective cohort study	Individual	12 mo
Dauzère et al 2017^18^	aTSA	Osteoarthritis, inflammatory arthritis, post-instability arthritis	Inclusion Perform pegged glenoid component	NR	NR	NA	not reported	35	Retrospective case-series	Shoulder	24 mo
DeVito et al 2019^19^	rTSA	Any indication except for acute fracture	NR	321	NR	NA	123 for SST, 120 for ASES	198 for SST, 196 for ASES	Retrospective case-series	Individual	24 mo
Flurin et al 2024^22^	aTSA or rTSA	Not specified	NR	165	0	NA	0	165	Prospective cohort study	Individual	12 mo
Hetto et al 2025^26^	aTSA	Osteoarthritis	Inclusion Aequalis Total Shoulder; Wright Medical, Memphis, TN, USA. Exclusion pre-operative rotator cuff tears, and neurological impairment of the treated shoulder	168	0	NA	30	138	Prospective cohort study	Individual	24 mo
Huber et al 2020^27^	rTSA	Unilateral cuff tear arthropathy Hamada grade ≥ 2	NR	203	10	6 deceased, 4 revisions	10 (25 for 24 mo ASES)	183 (168 for 24 mo ASES)	Prospective case-series	Individual	24 mo
Katz et al 2016^28^	rTSA	Irreparable massive cuff tear	NR	NR	NR	NA	NR	140 (134)	Retrospective case-series	Shoulder	24 mo
Kim et al 2024^29^	rTSA	Osteoarthritis, cuff tear arthropathy, irreparable massive cuff tear, proximal humerus fracture	Exclusion obvious complications following rTSA (eg, post-operative infection, periprosthetic fracture, instability, prosthetic failure), use of the ipsilateral upper extremity for weightbearing or transfer	95	10	Declined to participate	7	78	Retrospective case-series	Individual	12 mo
Lapner et al 2015^31^	aTSA	Osteoarthritis, inflammatory arthritis		87	7	NR	16	64	Retrospective case-series	Individual	24 mo
Lazaridou et al 2026^33^	aTSA or rTSA	Primary osteoarthritis, cuff tear arthropathy	Inclusion No missing values for dominance, 12-mo follow up data for at least one PROM, if patients had both sides treated only the first was included unless the second occurred at least one year after the first Exclusion incomplete pre-operative baseline data	2,592	260	No consent for research	180	2,152	Retrospective cohort study	Shoulder	12 mo
Mahony et al 2018^35^	aTSA	Osteoarthritis		699	0	NA	240	459	Retrospective case-series	Shoulder	24 mo
Mattiassich et al 2013^36^	rTSA	Proximal humerus fracture		32	5	Declined to participate, death, dementia, prosthesis explantation	11	16	Retrospective case-series	Individual	6 mo
McFarland et al 2021^37^	rTSA	Not specified	Inclusion patients receving social security disability insurance or worker’s compensation were matched to a control cohort	127	0	NA	2	125	Retrospective cohort study	Individual	24 mo
Mollon et al 2017^40^	rTSA	Any indication except for acute fracture	Exclusion infection, patients receiving constrained implants	660	0	NA	184	476 (474)	Case-control study	Shoulder	24 mo
Noble et al 2025a^42^	rTSA	Any indication		1,,098	0	NA	341	757	Retrospective case-series	Individual	6 mo
Noble et al 2025b^43^	rTSA	Any indication	Exclusion not high or low internal rotation	NR	NA	NA	NR	148	Retrospective case-series	Individual	24 mo
Pak et al 2024a^46^	rTSA	Any indication except for acute fracture	Inclusion reverse TSA with a 135 inlay humeral component	NR	NA	NA	NR	470	Case-control study	Individual	12 mo
Pak et al 2024b^47^	rTSA	Any indication except for acute fracture	Inclusion reverse TSA with a 135 inlay humeral component	NR	NA	NA	NR	442	Retrospective case series	Individual	12 mo
Raiss et al 2014^50^	aTSA	Osteoarthritis	Inclusion intact rotator cuff tear	63 (58)	16	Deceased	2	45 (40)	Prospective cohort study	Shoulder	15 y
Raiss et al 2018^51^	aTSA	Osteoarthritis	Inclusion cemented keeled glenoid component Exclusion previous infections or other diseases on the affected shoulder	126 (124)	2	1 revision, 1 deceased	10 individuals	118 (114)	Retrospective case-series	Shoulder	24 mo
Razmjou et al 2018^52^	aTSA	Osteoarthritis	Exclusion patients with ambidextrous limbs were not included in the analysis	NR	NA	NA	NR	152	Retrospective cohort study	Individual	12 mo
Schnetzke et al 2019^57^	aTSA	Osteoarthritis	Inclusion short-stem shoulder prosthesis	66 (64)	0	NA	1	65	Retrospective case-series	Shoulder	24 mo
Vegas et al 2023^63^	aTSA or rTSA	Not specified	Inclusion self-identified as playing a racket sport Exclusion unable to participate in racket sports for reasons other than their shoulder.	120	0	NA	81	39	Retrospective case-series	Individual	24 mo
White et al 2026^67^	aTSA	Glenohumeral osteoarthritis	Inclusion patients aged ≤65 yr at the time of surgery at least two yr out from surgery with complete ASES scores at baseline Exclusion ambidextrous hand dominance, bilateral surgery	308	0	NA	0	273	Prospective cohort study	Individual	24 mo

*aTSA*, anatomic total shoulder arthroplasty; *rTSA*, reverse total shoulder arthroplasty; *TSA*, total shoulder arthroplasty; *ROM*, range of motion; *ASES*, American Shoulder and Elbow Surgeons; *SST*, Simple Shoulder Test; *PROM*, patient-reported outcome measure.

**Table III:** Nonoutcome data.

Study ID	Group	Group size; n	Age; yr	Follow-up; mo	Sex; females n (%)	ASA status and comorbidities	Indication-specific characteristics	Smoking n (%)	DM; n (%)	Osteoporosis n (%)	Previous shoulder surgery n (%)	Other n (%)	BMI; kg/m^2^ or obesity rate	Additional procedures	Implant	Humeral fixation
Berthold et al 2020^5^	Dom	22	69.8 ± 7.2	36.5 ± 16.2	25 (62.5)	NR	Pre-operative glenoid version: 20.8 ± 8.4	NR	NR	NR	NR	NR	NR	NR	Unspecified (Arthrex)	Cementless
	Nondom	22	69.2 ± 7.9				Pre-operative glenoid version: 17.7 ± 7.1									
Cazeneuve et al 2011^9^	Dom	NR	75 (58-92)	86 (12-204)	33 (94)	Depression: 2 (5.7)	RCT: 6 (17.1) fracture: 24 (68.6) fracture dislocation 11 (31.4)	NR	5 (14.3)	NR	NR	Chronic alcoholism: 3 (8.6)	3 (8.6)	NR	NR	Cemented
	Nondom	NR														
Cazeneuve et al 2012^10^	Dom	NR	75 (58-92)	87.6 (12-204)	35 (94.6)	Depression: 2 (5.4)	NA	NR	5 (13.5)	NR	NR	Chronic alcoholism: 3 (8.1)	3 (8.1)	NR	Reverse Delta III shoulder prosthesis (DePuy-International Ltd)	Cemented
	Nondom	NR														
Cho et al 2021^11^	Dom	549	72.8 ± 7.4	31.6 ± 21.8	586 (74.5)	NR	NR	NR	NR	NR	NR	No work: 578 (73.4) light work: 109 (13.9) heavy work: 158 (20.1)	24.7 ± 3.8	NR	Medial glenoid-Medial humerus (Aequalis, TM Reverse Shoulder System, Delta Xtend, SMR): 384 (48.8) Medial glenoid-Lateral humerus: 270 (34.3) Lateral glenoid-Medial humerus: 59 (7) Lateral glenoid-Lateral humerus: 74 (9.4)	NR
	Nondom	238														
Collin et al 2017^12^	Dom	62	75.1 ± 6.0	24	71 (70.3)	NR	CTA: 59 (58.4) massive cuff tear: 19 (18.8) biconcave glenoid: 23 (22.8)	NR	NR	NR	NR	NR	26.5	NR	Aequalis Reversed (Wright Medical, Montbonnot, France)	Cemented
	Nondom	39														
Collin et al 2011^13^	Dom	31	66.7 (43-83)	120 (102-155)	31 (57.4)	NR	Glenoid morphology Walch and Boileau A1: 29 (51.8) A2: 1 (1.8) B1: 9 (16.1) B2: 15 (26.8) and C: 2 (3.6)	NR	NR	NR	NR	NR	NR	NR	Aequalis (Tornier, Mont Bonnot, France) Humeral head diameter: 39-50 mm glenoid curvature radii: 27.5, 30 or 32.5 mm	Cemented
	Nondom	25														
Cvetanovich et al 2015^17^	Dom	58	66.0 ± 9.1	23 (12-108)	25 (43.1)	NR	NA	NR	NR	NR	NR	NR	NR	NR	NR	NR
	Nondom	62	67.3 ± 10.2		33 (53.2)											
Dauzère et al 2017^18^	Dom	NR	67	27 (24-41)	22 (62.9)	NR	OA: 29 (82.9) inflammatory arthritis: 5 (14.3) post-instability arthritis: 1 (2.9) Glenoid morphology Walch and Boileau A: 26 (68.4) B: 12 (31.6)	NR	NR	NR	NR	NR	NR	NR	Perform pegged glenoid component (WrigthTornier, Inc., Edina, MN, USA) Humeral component Resurfacing head: 29 (76.3)anatomic short stem: 9 (23.7)	Cementless
	Nondom	NR														
DeVito et al 2019^19^	Dom	NR	75.2 ± 7.7	45 (24-133)	120 (60.6)	NR	NR	NR	NR	NR	NR	NR	NR	NR	DJO RSP (2007-2010) or DJO Monoblock RSP (2011-2015) RSA systems (DJO Surgical, Austin, TX, USA)	NR
	Nondom	NR														
Flurin et al 2024^22^	Dom	62	72.8 ± 6.6	12	103 (62)	ASA 1: 34 (21) ASA 2: 90 (54) ASA 3: 41 (25)	NR	NR	NR	NR	NR	Social isolation: 50 (30)	NR	NR	NR	NR
	Nondom	103														
Hetto et al 2025^26^	Dom	86	68.2 (51-85)	44.3 (24-123)	104 (75.4)	NR	NA	NR	NR	NR	NR	NR	NR	NR	Aequalis Total Shoulder (Wright Medical, Memphis, TN)	Cemented
	Nondom	52	69.5 (54-85)	46.8 (24-158)												
Huber et al 2020^27^	Dom	166	74.7 ± 6.5	24	122 (66.7)	ASA 1: 13 (7.1), ASA 2: 20 (10.9), ASA 3 66 (36.1), ASA 4+: 84 (45.9)	Hamada Grade 2: 49 (26.8) Grade 3: 41 (22.4) Grade 4+: 93 (50.8)	NR	NR	NR	NR	NR	NR	NR	Affinis inverse (Fa Mathys, Bettlach, Switzerland)	Cemented or cementless
	Nondom	17														
Katz et al 2016^28^	Dom	97	72 ± 6.9	45 ± 20	100 (74.6)	NR	Hamada Grade 2: 6 (4.3) Grade 3: 33 (23.6) Grade 4 73 (52.1) Grade 5 3 (2.1) Missing 23 (16.4) Walch classification type A: 55 (39.3) type B: 9 (6.4) type C: 2 (2.1) missing: 76 (54.3) 100	NR	NR	NR	NR	NR	NR	Biceps long head tenodesis: 67 (47.9)	Arrow universal shoulder arthroplasty (FH Orthopedics)	Cemented: 34 (24.3) cementless: 106 (75.7)
	Nondom	43														
Kim et al 2024^29^	Dom	42	70.8 ± 77.4	18.9 ± 7.3	29 (37)	NR	CTA: 25 (32.1) OA with intact rotator cuff: 19 (24.4) massive rotator cuff tear: 17 (21.8) osteoarthritis with rotator cuff tear: 13 (16.7%) proximal humerus fracture: 4 (5.0%).	NR	NR	NR	NR	NR	32.3 ± 6.7	NR	Perform glenoid component and Aequalis Ascend Flex humeral component (Stryker, Portage, MI)	NR
	Nondom	36														
Lapner et al 2015^31^	Dom	22	67 ± 11	24	36 (56.3)	NR	OA: 59 (90.7) RA: 5 (9.3)	NR	NR	NR	NR	NR	NR	NR	Press-fit humeral stem and keeled glenoid component (Tornier SAS, Saint-Ismier, France)	Cementless
	Nondom	42														
Lazaridou et al 2026^33^	Dom	1,404	73 ± 9	12	916 (65)	ASA 1: 49 (4) ASA 2: 721 (51) ASA 3: 622 (44) ASA 4: 11 (1)	OA: 452 (32) CTA: 952 (68) No rotator cuff tear: 243 (18) partial tear: 236 (17) single full tear or more: 893 (65)	126 (9)	NR	NR	NR	Alcohol 751 (54)	26.6 ± 4.8	Acromioplasty: 2 (0) Biceps tenodesis 594 (42) Muscle transfer: 32 (2) Glenoid bone graft: 67 (5)	Implant type reverse: 1,123 (80)	aTSA: cemented or cementless rTSA: cementless
	Nondom	748	73 ± 9		472 (63)	ASA 1: 33 (4) ASA 2: 366 (49) ASA 3: 336 (46) ASA 4: 10 (1)	OA: 323 (43) CTA 425 (57) Tear severity: No rotator cuff tear: 188 (26) partial tear: 163 (22) single full tear: 386 (52)	66 (9)				Alcohol 413 (56)	27.1 ± 5.1	Acromioplasty: 1 (0) Biceps tenodesis 410 (55) Muscle transfer: 6 (1) Glenoid bone graft: 38 (5)	Implant type reverse: 553 (74)	
Mahony et al 2018^35^	Dom	234	66.8 ± 9.3	13.2 (19.2-33.6)	205 (44.7)	ASA 1: 16(3.5) ASA 2: 352 (76.7) ASA 3+: 74 (16.1) Missing: 17 (3.7)	NA	NR	NR	NR	NR	NR	28.6 ± 5.6	Subscapularis tendor repair and biceps tenodesis as covariates to the model but no aggregate data	Biomet Comprehensive (Warsaw, IN, USA)	NR
	Nondom	174														
Mattiassich et al 2013^36^	Dom	10	72 ± 8	20 (6-42)	27 (84.4)∗ although not reported for the 16 in the study	NR	Neer classification 4-part fractures: 15 (93.8) 3-part fractures: 1 (6.3)	NR	NR	NR	NR	NR	28.9	Refixation of greater and lesser trochanters in all cases	Delta Xtend (DePuy-Johnson&Johnson, Warsaw, IN)	Cemented
	Nondom	6														
McFarland et al 2021^37^	Dom	62	63.2	35.7 (24-96)	76 (60.8)	Cardiovascular: 32 (25.6) depression: 24 (19.2) liver disease: 12 (9.6) neurological disease: 1 (0.8) respiratory disease: 3 (2.4)	NR	35 (28.0)	12 (9.6)	NR	4 (3.2)	Sedentary: 21 (16.8) light work: 30 (24.0) heavy work: 62 (49.6) retired after shoulder injury: 8 (6.4) disabled due to injury: 4 (3.2) married: 63 (50.4) college education: 74 (59.2)	Obese: 48 (38.4) morbidly obese: 13 (10.4)	NR	January 1, 2009, to December 3, 2014, Reverse Shoulder Prosthesis (DJO, Austin, TX): 42 (33.6) January 4, 2014, to December 12, 2016, ReUnion RSA (Stryker, Mahwah, NJ): 83 (66.4)	Cemented: 42 (33.6) Cementless: 83 (66.4)
	Nondom	63														
Mollon et al 2017^40^	Dom	313	72.5 (53-90)	38 (22-93)	312 (66)	NR	NR	NR	NR	NR	NR	NR	27.8 ± 5.3	Latissimus dorsi transfer: 3 (0.6) subscapularis repair: 259 (54.4) baseplate screws: 4.2 ± 0.5	Equinoxe rTSA (Exactech, Inc., Gainesville, FL) 38-expanded glenosphere 38-mm x 25-mm: 189 (39.7) 42-mm x 27-mm: 11 (2.3) standard glenosphere 38-mm x 21-mm: 256 (53.8) 42-mm x 23-mm: 10 (2.1) 46-mm x 25-mm: 10 (2.1)	NR
	Nondom	163														
Noble et al 2025a^42^	Dom	439	72.4 ± 8.0	23.9 ± 19.9	424 (66)	Renal disease: 85 (11.2)	Inflammatory arthritis:60 (7.9) OA 163 (21.5) CTA/irreparable rotator cuff tear: 469 (62.0) proximal humerus fracture/sequela: 113 (14.9) other: 12 (1.6)	81 (10.7)	300 (39.6)	49 (6.5)	256 (33.8)	NR	29.0 ± 6.1	NR	NR	NR
	Nondom	318														
Noble et al 2025b^43^	Dom	100	68.5 ± 7.7	24	78 (52.7)	NR	NR	7 (4.7)	14 (8.4)	NR	NR	NR	29.2 ± 5.1	NR	Univers Revers; Arthrex, Inc.; Universal Baseplate; Arthrex, Inc before 4/2018, Modular Glenoid System; Arthrex, Inc. after 4/2018 Glenosphere diameter 33: 29 (19.6) 36: 46 (31.1) 39: 47 (41.8) 42: 28 (18.1) lateralization 0-2 mm: 10 (6.8) 4 mm: 26 (17.6) 6 mm: 60 (40.5) 8 mm: 54 (36.5)	NR
	Nondom	48														
Pak et al 2024a^46^	Dom	275	69.9 ± 7.4	12	226 (48.1)	NR	CTA: 258 (54.9) irreparable rotator cuff tear: 13 (2.8) OA: 251 (53.4) pre-operative acromial thinning: 21 (4.5)	23 (4.9)	NR	NR	47 (10.0)	NR	30.3 ± 6.3	Subscapularis tendon repair: 186 (39.6)	Univers Revers; Arthrex, Inc.; Universal Baseplate; Arthrex, Inc before 4/2018, Modular Glenoid System; Arthrex, Inc. after 4/2018 Glenosphere diameter 33: 29 (19.6) 36: 46 (31.1) 39: 47 (41.8) 42: 28 (18.1) lateralization 0-2 mm: 10 (6.8) 4 mm: 26 (17.6) 6 mm: 60 (40.5) 8 mm: 54 (36.5)	NR
	Nondom	195														
Pak et al 2024b^47^	Dom	255	70 ± 7.5	12	211 (47.7)	NR	NR	NR	48 (10.9)	NR	NR	NR	30.3 ± 6.4	NR	Univers Glenosphere diameter 33: 29 (19.6) 36: 46 (31.1) 39: 47 (41.8) 42: 28 (18.1) lateralization 0-2 mm: 10 (6.8) 4 mm: 26 (17.6) 6 mm: 60 (40.5) 8 mm: 54 (36.5)	NR
	Nondom	187														
Raiss et al 2014^50^	Dom	36	64 (44-80)	15+ yr	12 (26.7)	NR	NA	NR	NR	NR	NR	NR	NR	NR	Aequalis (Tornier, Mont Bonnot, France)	Cemented
	Nondom	9														
Raiss et al 2018^51^	Dom	NR	68 (51-85)	38 (24-70)	63 (55.3)	NR	NA	NR	NR	NR	NR	NR	NR	Biceps tenodesis and subscapularis tendon repair in all cases	Keeled glenoid component (Wright Medical, Memphis, TN, USA)	NR
	Nondom	NR														
Razmjou et al 2018^52^	Dom	80	60	12	87 (57.2)	NR	NA	NR	NR	NR	NR	Bilateral symptoms: 67 (44.1) unilateral symptoms: 85 (55.9)	NR	NR	NR	NR
	Nondom	72														
Schnetzke et al 2019^57^	Dom	35	70 (47-85)	37 (24-58)	38 (58)	NR	Glenoid morphology Walch and Boileau A1: 9 (13.8) A2: 37 (56.9) B1: 5 (7.7) B2: 13 (20.0) and B3: 1 (1.5)	NR	NR	NR	NR	NR	NR	NR	Ascend Flex (Wright Medical, Memphis, TN, USA)	Cementless
	Nondom	30														
Vegas et al 2023^63^	Dom	22	70 [65.0-76.4]	38 [30.5-56.5]	10 (25.6)	NR	NR	NR	NR	NR	NR	NR	NR	NR	Standard or short humeral stemmed TSA (DJO Turon or Altivate Anatomic, Austin, TX) or RSA (DJO RSP, Austin, TX)	NR
	Nondom	17														
White et al 2026^67^	Dom	95	57.9 ± 5.3	33.6 ± 20.4	17 (17.9)	CCI: 0.4 ± 0.9	Overhead sports yes 53 (55.8)	NR	NR	NR	NR	NR	27.8 ± 4.5	NR	NR	NR
	Nondom	70	58.3 ± 6.1		19 (27.1)	CCI: 0.3 ± 0.5	Overhead sports yes 33 (47.1)						28.2 ± 4.3			

*ASA*, American Society of Anesthesiologists; *DM*, diabetes mellitus; *BMI*, body mass index; *OA*, osteoarthritis; *RCT*, randomized controlled trial; *CTA*, cuff tear arthropathy; *RA*, rheumatoid arthritis; *aTSA*, anatomic total shoulder arthroplasty; *rTSA*, reverse total shoulder arthroplasty; *TSA*, total shoulder arthroplasty; *CCI*, Charlson-Deyo Comorbidity Index; *dom*, dominant.

### Risk of bias in studies

**Figure 2:**
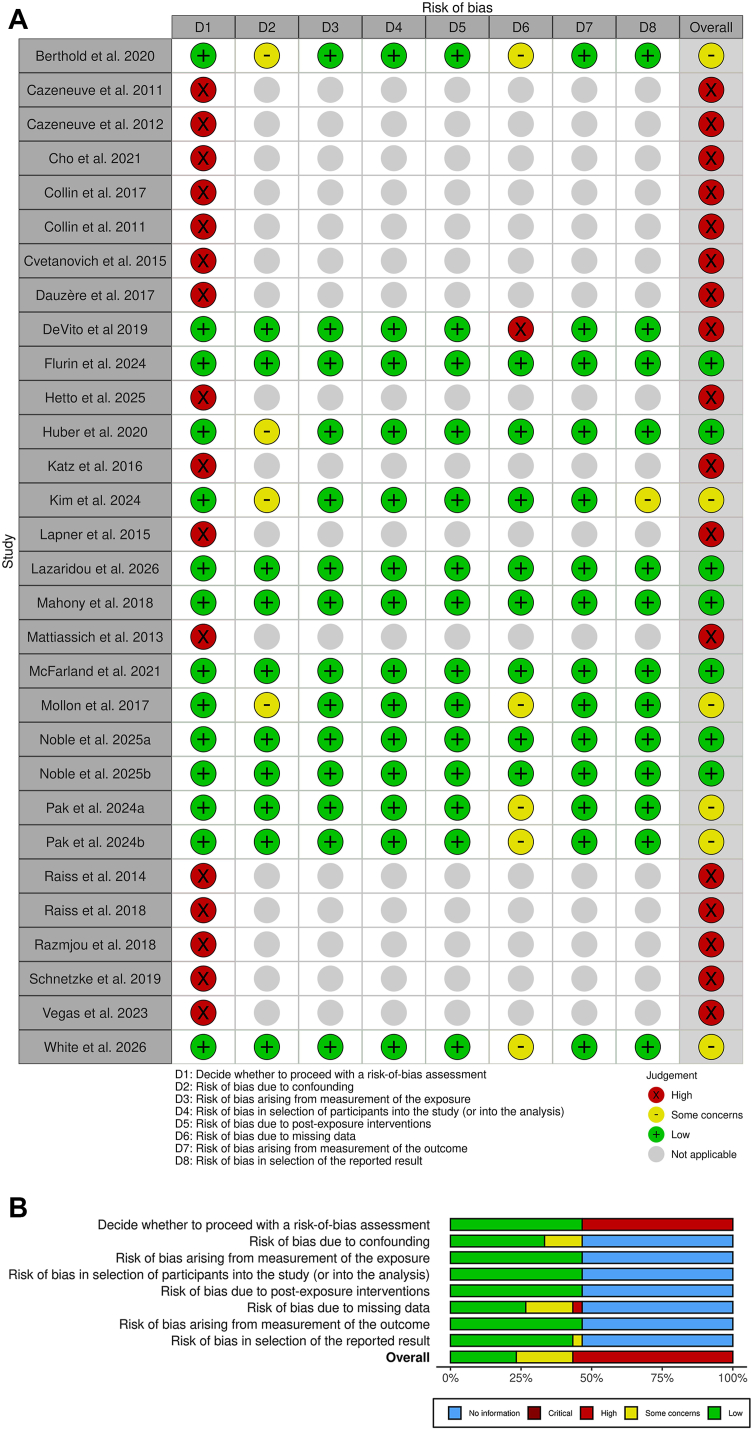
(**A**) Traffic-light plot. (**B**) Summary plot.

Risk of bias assessment is presented in Figure 2. Seventeen studies^9^, ^10^, ^11^, ^12^, ^13^^,^^17^^,^^18^^,^^26^^,^^28^^,^^32^^,^^36^^,^^50^, ^51^, ^52^^,^^57^^,^^63^ were characterized as high risk-of-bias, six^5^^,^^29^^,^^40^^,^^46^^,^^47^^,^^67^ as some concerns of bias, and seven^22^^,^^27^^,^^33^^,^^35^^,^^37^^,^^42^^,^^43^ as low risk-of-bias. Sixteen studies^9^, ^10^, ^11^, ^12^, ^13^^,^^17^^,^^18^^,^^26^^,^^28^^,^^32^^,^^36^^,^^50^, ^51^, ^52^^,^^57^^,^^63^ were considered to be at high risk of bias without requiring a detailed risk-of-bias assessment due to substantial potential for confounding in the unadjusted result. The study by DeVito et al^19^ was considered to have an overall high risk-of-bias due to missing data.

### Results of syntheses

#### Physical examination and symptoms

Three studies (300 shoulders, 300 patients) reported baseline forward elevation—two after aTSA and 1 after rTSA—with no differences being noted for both procedures.^5^^,^^17^^,^^26^ Four studies (401 shoulders, 401 patients) assessed post-operative forward elevation.^5^^,^^12^^,^^17^^,^^26^ Collin et al 2017^12^ found that at 6 months after rTSA, the incidence of forward elevation <90° was higher in the nondominant group (Table IV). For aTSA, Cvetanovich et al^17^ found a difference in favor of the dominant group at mean 23 (range, 12-108) months, while Berthold et al^5^ and Hetto et al^26^ showed no differences (Table IV).

**Table IV:** Summary of continuous range of motion comparisons.

Study ID	Follow-up; mo	Side	Forward elevation; degrees	*P* value	Abduction; degrees	*P* value	External rotation; degrees	*P* value	Internal rotation; degrees	*P* value
Berthold et al^5^	36.5 ± 16.2	Dominant	151.2 ± 28.3	.76	142.7 ± 33.1	.96	52.2 ± 17.3	.74	NR	NR
		Nondominant	138.1 ± 41.3		134.1 ± 45.9		49.7 ± 22.7			
Cvetanovich et al^17^	23 (range, 12-108)	Dominant	151, range 146-155	**.033**	NR	NR	61, range 58-65	**.001**	52, range 58-65	.419
		Nondominant	141, range 134-149				51, range 48-55		51, range 48-55	
Hetto et al^26^	45 (range, 24-158)	Dominant	149.2	n.s.	142.2	n.s.	43.3	n.s.	NR	NR
		Nondominant	151.3		141		42.7			

Bold indicates statistical significance.

Two studies (180 shoulders, 180 patients) assessed post-operative abduction in patients after aTSA with similar baseline measurements, and no differences were noted between groups.^5^^,^^26^ External rotation after aTSA was reported by 3 studies (300 shoulders, 300 patients) with similar pre-operative measurements among groups.^5^^,^^17^^,^^26^ Cvetanovich et al^17^ found a difference in favor of the dominant group, while Berthold et al^5^ and Hetto et al^26^ did not (Table IV).

Two studies (268 shoulders, 268 patients) assessed post-operative internal rotation.^17^^,^^43^ Cvetanovich et al^17^ found no differences between groups after aTSA, but Noble et al^43^ reported that the dominant group was associated with higher odds of high internal rotation (T12 or better vs. low; hip or below; adjusted odds ratio [OR]: 5.4, 95% confidence interval [CI]: 1.3-22.1; *P*= .019) after rTSA. Return of strength after aTSA was reported by two studies (202 shoulders, 202 patients), and no differences were observed.^26^^,^^32^ Razmjou et al^52^ described no difference in incidence of bilateral symptoms at 1 year after aTSA (dominant vs. nondominant, 42.5% vs. 48.5%; *P*= .68).

#### Patient-reported outcome measures

Six studies (2,656 shoulders, 2,656 patients) assessed pre-operative VAS pain scores.^5^^,^^17^^,^^22^^,^^26^^,^^33^^,^^63^ Vegas et al^63^ reported increased baseline VAS score for the dominant arm. Five studies assessed post-operative pain using VAS.^5^^,^^17^^,^^22^^,^^26^^,^^63^ Flurin et al^22^ reported that arm dominance was not a factor for pain at 6 months (adjusted OR: 1.3, 95% CI: 0.6-2.7; *P*= .579) or 1 year (adjusted OR: 1.2, 95% CI: 0.6-2.5; *P*= .628), irrespective of procedure. For both procedures, no difference was reported by the other 5 studies (Table V).^5^^,^^17^^,^^26^^,^^33^^,^^63^ Lazaridou et al^33^ reported that at 12 months after aTSA and rTSA, dominance was not associated with a difference in the odds of achieving Minimal Clinically Important Difference (MCID) or Patient Acceptable Symptom State (PASS) for pain.

**Table V:** Summary of continuous PROMs comparisons.

Study ID	Follow-up; mo	Side	Pain; VAS	*P* value	ASES	*P* value	SST	*P* value	CMS	*P* value
Berthold et al.^5^	36.5 ± 16.2	Dominant	2.4 ± 3.3	.83	77.3 ± 24	.77	8.8 ± 2.9	.96	NR	NR
		Nondominant	1.8 ± 3.1		78.8 ± 29.5		8.4 ± 3.6			
Cvetanovich et al.^17^	23 (range, 12-108)	Dominant	0.9, 95% CI: 0.6-1.3	.129	84, 95% CI: 81-88	.211	9.8, 95% CI: 9.2-10.3	.278	NR	NR
		Nondominant	1.4, 95% CI: 0.9-1.9		80, 95% CI: 76-85		9.2, 95% CI: 8.5-10.0			
Hetto et al.^26^	45 (range, 24-158)	Dominant	1.3, range 0.2-1.5	n.s.	NR	NR	NR	NR	66.1, range 10-88	.8
		Nondominant	1.3, range 0.5-1.5						65.8, range 21-85	
Lazaridou et al.^33^	12 mo	Dominant	1.3 ± 2.0	NR	NR	NR	NR	NR	32 ± 14	NR
		Nondominant	1.3 ± 2.0						33 ± 15	
Razmjou et al.^52^	12 mo	Dominant	NR	NR	81 ± 16	.99	NR	NR	83 ± 22	.52
		Nondominant			80 ± 14				86 ± 21	
Schnetzke et al.^57^	37 (range, 24-58)	Dominant	NR	NR	NR	NR	NR	NR	76 ± 10	.149
		Nondominant							72 ± 13	
Vegas et al.^63^	38 [30.5-56.5]	Dominant	0 [0-2]	.968	NR	NR	NR	NR	NR	NR
		Nondominant	0 [0-2]							
White et al∗^67^	33.6 ± 20.4	Dominant	NR	NR	39.1 ± 21.7	.11	NR	NR	NR	NR
		Nondominant			45.4 ± 22.9					

*PROM*, patient-reported outcome measure; *CI*, confidence interval; *VAS*, visual analog scale; *ASES*, American Shoulder and Elbow Surgeons; *SST*, Simple Shoulder Test; *CMS*, Constant Murley score.

∗ Change from baseline.

Pre-operative ASES was assessed by 4 studies (587 shoulders, 587 patients), with no differences being reported for both procedures.^5^^,^^17^^,^^52^^,^^67^ Post-operative ASES was examined by 8 studies (1,241 shoulders, 1,241 patients).^5^^,^^17^^,^^19^^,^^22^^,^^27^^,^^37^^,^^52^^,^^67^ Flurin et al^22^ reported that arm dominance was not a factor for ASES at 6 months (adjusted OR: 0.2, CI: 0.01-200.3; *P*= .666) or 1 year (adjusted OR: 2.5, CI: 0.01-897.9; *P*= .757), irrespective of procedure. Huber et al^27^ defined treatment effect as $\frac{\left|postoperative-preoperative\;score\right|}{preoperative\;score}$, and reported that dominance was not a factor (OR: 0.9, 0.8-1.0; *P*= .07) at 2 years after rTSA. No differences were reported by the other included studies for both procedures (Table V).^5^^,^^17^^,^^52^^,^^67^ DeVito et al^19^ reported that arm dominance was not a factor (OR: 0.6; *P*= .99) for ASES at mean 45 (range, 24-158) months after rTSA. Similar results were found by McFarland et al^37^ at mean 35.7 (24-96) months after rTSA. Finally, Berthold et al^5^ found no in achievement rates for PASS (dominant vs. nondominant, 50% vs. 71%; *P*= .3), MCID (dominant vs. nondominant, 85% vs. 93%; *P*= .58), and Substantial Clinical Benefit (dominant vs. nondominant, 54% vs. 73%; *P*= .43) after aTSA.

Two studies (162 shoulders, 162 patients) assessed preoperative SST, and no differences were reported.^5^^,^^17^ Post-operative SST was reported by 3 studies (360 shoulders, 360 patients).^5^^,^^17^^,^^19^ Berthold et al^5^ and Cvetanovich et al^17^ reported no differences after aTSA, while DeVito et al^19^ reported that dominant shoulders had lower odds of achieving maximal clinical change, $\frac{postoperative-preoperative\;score}{maximum\;postoperative-preoperative\;score}$, (adjusted OR: 0.4; *P*= .002) after rTSA.

Seven studies assessed post-operative CMS (2,733 shoulders, 2,728 patients).^22^^,^^26^^,^^33^^,^^36^^,^^50^^,^^52^^,^^57^ Flurin et al^22^ reported that arm dominance was not a factor for CMS at 6 months (adjusted OR: 1.3, 95% CI: 0.02-99.5; *P*= .845) or 1 year (adjusted OR: 3.7, 95% CI: 0.2-90.0; *P*= .423). Razmjou et al^52^ also found no difference in CMS at 1 year. No difference was reported by Hetto et al.,^26^ Mattiassich et al^36^ (*P*= .051), and Schentzke et al^57^ at midterm follow-up, or by Raiss et al^50^ at 15-year follow-up. Lazaridou et al^33^ reported that at 12 months after aTSA and rTSA, dominance was not associated with a difference in the odds of achieving MCID or PASS for CMS.

Finally, at 6-month and 1-year follow-up after rTSA, Flurin et al^22^ reported that arm dominance was not associated with SPADI (6 months; OR: 0.1 95% CI: 0.01-270.4, *P*= .489, 1 year; OR: 0.4 95% CI: 0.01-1,638.3, *P*= .831) and University of California and Los Angeles scores (6 months; OR: 0.6 95% CI: 0.1-4.1, *P*= .622, 1 year; OR: 2.2 95% CI: 0.5-9.0, *P*= .309). Lazaridou et al^33^ reported that at 12 months after aTSA and rTSA, dominance was not associated with a difference in the odds of achieving MCID or PASS for SPADI and QuickDASH.

#### Satisfaction, independence, and activities of daily living

Three studies (489 shoulders, 489 patients) assessed post-operative satisfaction; two after aTSA^26^^,^^67^ and 1 after rTSA.^29^ Hetto et al^26^ assessed satisfaction after aTSA on a Likert-like scale and found no difference in percentage of patients assessing their results as “good” or “very good” (dominant vs. nondominant, 91.9% vs. 92.3%; *P*= .83). Similarly, White et al^67^ examined satisfaction at a minimum 24 months after aTSA and found no difference between dominant and nondominant side procedures (80% vs. 91%, *P*= .051). Kim et al^29^ examined internal rotation as a factor for patient satisfaction (yes or no) at minimum 12 months after rTSA. Overall odds for satisfaction were not statistically significantly different (OR: 0.7, 95% CI: 0.3-1.6; *P*= .364), but dissatisfaction was more likely when internal rotation was impaired on the dominant side (dominant vs. nondominant, 68% vs. 35%, *P*= .02). Among patients who underwent surgery on their dominant side, toileting ability was significantly more limited when internal rotation was restricted to the ipsilateral side or buttock level (*P*= .021).

Two studies by Cazeneuve et al^9^^,^^10^ (37 shoulders, 35 patients) reported on independency after rTSA. No loss of autonomy was described for nondominant side procedures. For the dominant group, a loss of autonomy or a major increase in dependency was observed for 28% of cases.^9^ In addition, 41% of dominant cases reported being unable to use eating utensils, to dress independently, or to perform activities necessary for personal hygiene at mean 87.6 (range, 12-204) months after rTSA.^10^

#### Radiographic findings

Three studies (209 shoulders, 203 patients) assessed RLLs after aTSA.^13^^,^^18^^,^^51^ Both Dauzère et al^18^ and Raiss et al^51^ found no correlation between RLLs and dominant side at mean 27 (range, 24-41) months and 38 (range 24-70) months, respectively. Collin et al^13^ found that protheses implanted in the dominant limb were associated with a significantly higher rate of RLL development (*P*= .017) at 10-year follow-up.

#### Complications and revision surgery

Three studies (2,014 shoulders, 2,014 patients) assessed ASF incidence after rTSA.^11^^,^^42^^,^^46^ Pak et al^46^ showed that at 1 year, the incidence of ASF was higher for dominant arms (adjusted OR: 2.2, 95% CI: 1.3-5.8; *P*= .037). Cho et al^11^ found no difference in combined incidence of acromial or scapular spine stress fractures (3.8% vs. 3.4%; *P*= .751) at mean 31.6 ± 21.8 months of follow-up. Noble et al^42^ reported that arm dominance was not associated with ASF incidence (8.2% vs. 7.3%; *P*= .651) in unadjusted results and when stratified by sex. In multivariable analysis, surgery on the dominant side was associated with increased risk for ASF (OR: 1.9, 95% CI: 1.2-4.9; *P*= .010).

Scapular and subacromial notching were reported by 3 studies (1,058 shoulders, 1,052 patients) after rTSA.^28^^,^^40^^,^^47^ At mean 45 ± 20 months post-operatively, Katz et al^28^ showed that scapular notching was more likely on the dominant side compared to the nondominant (83% vs. 17%, *P*= .04). Mollon et al^40^ reported opposite results (8.0% vs. 14.1%; *P*= .036) at mean 38 (range, 22-93) months post-operatively. Pak et al^47^ showcased that at 1 year, the incidence of subacromial notching (adjusted OR: 0.3, CI: 0.2-4.2; *P*= .646) was not affected by arm dominance.

At 1-year follow-up, Flurin et al^22^ reported that arm dominance was not a factor for complications (defined as combined hematoma, acromial fracture, instability, Surgical Site Infection; *P*= .604), irrespective of procedure. Hetto et al^26^ found no statistically significant differences in revision surgery rates (1.2% vs. 1.9%; *P*= .89) after aTSA. Mahony et al^35^ defined as failed aTSA the need for revision or not meeting the MCID threshold on ASES at a mean 13.2 (range, 19.2-33.6) months. They reported lower adjusted odds for failure on the nondominant side (adjusted OR: 0.3, CI: 0.1-0.7; *P*= .007), with similar revision rates among those who failed (33.3% vs. 30.0%, *P*= .286).

## Discussion

### Interpretation

**Figure 3:**
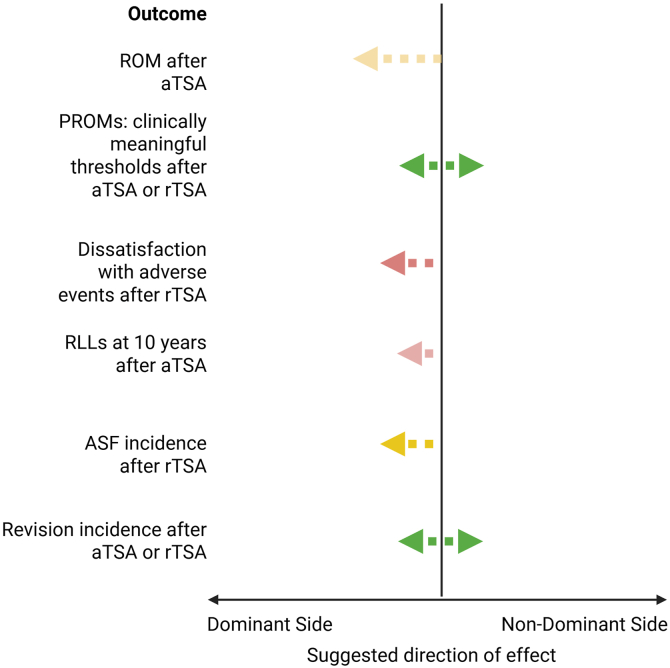
Summary of observed patterns. Color represents overall risk-of-bias; red = high risk-of-bias, yellow = some concerns of bias, green = low risk-of-bias. Transparency represents number of studies, with increased transparency indicating less studies. Size of arrow represents suggested effect size. Created with Biorender. *ROM*, range of motion; *aTSA*, anatomic total shoulder arthroplasty; *rTSA*, reverse total shoulder arthroplasty; *PROM*, patient-reported outcome measure; *ASF*, acromial stress fracture; *RLL*, radiolucent line.

To the best of our knowledge, this is the first systematic review to assess the effect of arm dominance on aTSA and rTSA outcomes, and certain patterns that could inform patient–physician expectations and future research emerged (Fig. 3). Specifically, aTSA on the dominant side might be associated with better and earlier return of post-operative ROM. Nevertheless, this potential early functional difference is probably not associated with a different likelihood of achieving clinically meaningful thresholds in PROMs, irrespective of procedure. Limb dominance is not associated with satisfaction after aTSA, but dominant-side rTSA could perhaps be associated with a higher risk of dissatisfaction with adverse events. The dominant side might be associated with increased incidence of RLLs at long-term follow-up after aTSA and increased incidence of ASF after rTSA. Irrespective of procedure, there was no evidence to suggest an association of limb dominance with other post-operative complications or revision surgery.

Our first aim was to examine ROM post-operatively. Several studies with similar pre-operative baselines among groups showed superior post-operative ROM in dominant limb after aTSA.^12^^,^^17^^,^^43^ Most of the comparisons came from a single study on aTSA with high risk of bias,^17^ although the study by Noble et al^43^ provided adjusted results with the same direction of effect but for rTSA. The rest of the studies on aTSA were later in the post-operative course and yielded nonstatistically significant results,^5^^,^^26^ but the direction of effect was in favor of the dominant limb consistently. While not statistically proven, the consistent pattern of the results suggests that patients with surgery on the dominant arm might achieve ROM milestones earlier in the postoperative course, particularly after aTSA.

Our second aim was to assess PROMs. This was the most commonly reported outcome measure among the included studies, with 14 of the studies assessing at least one PROM.^5^^,^^17^^,^^19^^,^^22^^,^^26^^,^^27^^,^^33^^,^^36^^,^^37^^,^^50^^,^^52^^,^^57^^,^^63^^,^^67^ Irrespective of procedure, no statistically significant difference was noted between dominant and nondominant arms for any post-operative PROM comparison at any length of follow-up. Interestingly, while 12 studies assessed PROMs, only 4 assessed them in the context of clinically meaningful thresholds instead of just comparing post-operative scores. After adjusting for confounders, DeVito et al^19^ reported that patients having TSA on their dominant arm had a statistically significantly worse likelihood of achieving maximal clinical change on SST. Huber et al^27^ and Berthold et al^5^ showed no difference in treatment effect and likelihood of PASS, MCID, and substantial clinical benefit, although both studies were underpowered. Nevertheless, a recent study by Lazaridou et al,^33^ including 2,152 shoulders—the largest sample size in our study—found that arm dominance was not meaningfully associated with the likelihood of achieving MCID or PASS for SPADI, QuickDASH, VAS, or CMS after aTSA or rTSA. Taken together, the available evidence suggests that arm dominance is unlikely to be a determinant of patient-perceived clinically meaningful improvement following aTSA or rTSA.

This pattern is supported by the results observed for our third aim assessing satisfaction, independence, and activities of daily living. Even though these outcomes were assessed by a small number of studies with heterogeneous populations and methodological limitations, the results were again consistent. Satisfaction, independence, and activities of daily living were not affected by surgery laterality irrespective of procedure.^9^^,^^10^^,^^26^^,^^29^ Nevertheless, the two studies by Cazeneuve et al^9^^,^^10^ suggest that poor outcomes after rTSA were more likely cause dissatisfaction and loss of independence in dominant-side procedures. Given the increased use of the dominant hand in everyday activities, this finding seems intuitive and should be further explored.

Our fourth aim was to assess post-operative radiographic findings potentially indicative of long-term complications. Studies assessing presence of RLLs after aTSA had notable limitations, but their combined results suggest a pattern. A study evaluating midterm follow-up results showed no difference in RLLs around the glenoid component,^18^^,^^51^ while Collin et al^13^ showed that at 10 years, implants on the dominant side were more likely to show RLLs. Most midterm follow-up studies evaluating RLLs after aTSA suggest that they are linked to factors such as implant positioning, glenoid deformity, and surgical technique rather than post-operative activity level, which would likely be higher in the dominant arm.^20^^,^^48^^,^^53^^,^^65^ Perhaps, these higher activity levels may contribute to a higher rate of RLLs at long-term follow-up by amplifying subtle differences in implant alignment and surgical technique.

Finally, we examined post-operative complications. Pak et al^46^ reported that rTSA on the dominant side had twice the likelihood of ASF incidence at 1 year, after adjusting for all known patient-related and technique-related confounders.^41^^,^^55^^,^^61^^,^^70^ Using a similar model, Noble et al^42^ showed that arm dominance was associated with ASF incidence at two years, but the association was not significant after adjustment for multiple hypotheses testing. Cho et al^11^ had a substantial sample size and did not observe a statistically significant difference in unadjusted ASF rates. Handedness was not included in their multivariable model; and therefore, the true impact of arm dominance cannot be fully elucidated. These findings are intuitive and likely based on increased usage of the dominant arm relative to the nondominant, which likely increased the stress placed on the acromion.

The evidence was conflicting with respect to subscapular and subacromial notching after rTSA. Pak et al^47^ showed no difference in adjusted odds at 1 year, while Katz et al^28^ and Mollon et al^40^ showed statistically significant differences but with opposite directions of effect, yielding no clear consensus. In contrast, all studies evaluating complications and revision surgery risk showed that arm dominance is not related to other post-operative complications and incidence of revision surgery, irrespective of procedure.^22^^,^^26^^,^^35^

### Limitations

The most notable limitation of the included studies was the lack of adjustment for confounders. This can be partially explained by the fact that handedness was not the primary comparison of interest in most of the studies; therefore, adjustments specific to that comparison were not made in otherwise well-designed studies. However, studies that primarily examined this comparison did not report adjusted results and had limited clinical data to allow for the evaluation of the impact of confounding factors in the results. In addition, implant designs have changed over time; therefore, certain outcomes, such as scapular notching, have lower incidence in newer studies.^47^^,^^69^ This could partially explain the heterogeneity observed in incidence rates and in direction of effect, as there was a mix of Grammont-style designs and newer designs with a 135° neck-shaft angle and lateralized glenosphere or humeral components. Finally, several studies analyzed shoulders rather than patients without accounting for the clustered nature of the data, which may have led to unit-of-analysis errors and underestimated variance. This could be an important confounder in comparison of operative sides, as patients might report worse outcomes for their first surgery while experiencing symptoms on the nonoperated side.^62^ Other limitations of the included studies included that the majority were retrospective in design and had limited sample size, loss of follow-up, and lack of a designated control group. Importantly, there was heterogeneity in the surgical indications and in their severity among studies, which was one of the primary reasons why we decided not to perform a meta-analysis. Although meta-analysis allows quantitative synthesis and may strengthen confidence in the findings, it was not feasible in this review because of heterogeneity among the included studies and concerns identified in the risk-of-bias assessment. Therefore, the proposed hypotheses should be interpreted with appropriate caution and examined by future studies. Nevertheless, this study provides a comprehensive qualitative synthesis of the available evidence that shoulder arthroplasty surgeons could use to inform pre-operative patient counseling.

## Conclusion

This systematic review is the first to examine the influence of limb dominance on outcomes after TSA, an aspect often recorded but rarely studied in depth. Even though the available evidence has notable limitations and no quantitative synthesis was performed, by consolidating and critically appraising the existing evidence, our study found consistent patterns with clinical significance to post-operative recovery. Greater use of the dominant arm, particularly after aTSA, could facilitate earlier achievement of functional milestones; yet, it may also predispose to long-term radiographic changes such as RLLs. Despite potential earlier functional gains, evidence suggests that arm dominance is not associated with differences in patient-perceived clinically meaningful improvement, irrespective of procedure. Notably, our synthesis suggested a potential association between dominance and early incidence of ASF after rTSA. Our findings offer clinically relevant patterns that could be useful for patient counseling and as hypotheses for future research that addresses this important evidence gap.

## Disclaimers:

Funding: Periklis Giannakis' and Jashvant Poeran's research salary is funded by the Anesthesiology Departmental Mission Fund, Department of Anesthesiology, Critical Care & Pain Management, Hospital for Special Surgery.

Conflicts of interest: Jashvant Poeran owns stock options at Parvizi Surgical Innovations.

Robert G. Marx reports a relationship with International Society of Arthroscopy Knee Surgery and Orthopedic Sports Medicine that includes board membership, a relationship with Mend Nutrition Inc that includes equity or stocks and has a patent with royalties paid to Springer and Demos Health.

Samuel A. Taylor reports a relationship with Enovis/Don Joy Orthopedics that involves consulting/honorarium and royalties, a relationship with Smith and Nephew that involves consulting and a relationship with Arthrex that involves consulting.

Lawrence V. Gulotta has a relationship with Zimmer-Biomet, Inc. that includes speaking, consulting, and royalties.

Any additional authors, their immediate families, and any research foundations with which they are affiliated have not received any financial payments or other benefits from any commercial entity related to the subject of this article.
