# Supplementary material for: Does upper limb dominance influence outcomes in adults undergoing primary anatomic or reverse total shoulder arthroplasty? A systematic review
Source: J Shoulder Elb Arthroplast. 2026 Apr 21;10(3):100023. doi: 10.1016/j.jsea.2026.100023 (PMC13264064; doi:10.1016/j.jsea.2026.100023)
Supplement: Supplementary Material [file mmc1.docx]

**Supplementary table 1. Line-by-line search strategy using the PICO framework**

| **Bibliographic database** | **Search prompt** |
| --- | --- |
| PubMed | ("Arthroplasty, Replacement, Shoulder"[Mesh] OR "Shoulder Prosthesis"[Mesh] OR “Shoulder Arthroplasty” OR “Shoulder Replacement” OR “Anatomic Total Shoulder Arthroplasty” OR “Anatomic Total Shoulder Replacement” OR “Conventional Total Shoulder Arthroplasty” OR “Reverse Total Shoulder Arthroplasty” OR “Reverse Total Shoulder Replacement” OR TSA OR rTSA or “primary TSA*”) AND (“Upper Extremity Dominance” OR “Upper Limb Dominance” OR “Hand Dominance” OR “Arm Dominance” OR “Dominant Hand” OR “Non-Dominant Hand” OR “Nondominant Hand” OR “Dominant Upper Limb” OR “Non-Dominant Upper Limb” OR “Nondominant Upper Limb” OR “Dominant Limb” OR “Non-Dominant Limb” OR “Nondominant Limb” OR “Dominant Upper Extremity” OR “Non-Dominant Upper Extremity” OR “Nondominant Upper Extremity” OR “Dominant Extremity” OR “Non-Dominant Extremity” OR “Nondominant Extremity” OR “ Dominant Arm” OR “Non-Dominant Arm” OR “Nondominant Arm” OR “Dominant-Arm” OR “Nondominant-Arm” OR “Non-Dominant-Arm” OR “Dominant Group” OR “Non-Dominant Group” OR “Nondominant Group” OR “Dominant Side” OR “Non-Dominant Side” OR “Nondominant Side” ) |
| Embase | ('shoulder arthroplasty'/exp OR 'shoulder prosthesis'/exp OR 'shoulder arthroplasty' OR 'shoulder replacement' OR 'anatomic total shoulder arthroplasty' OR 'anatomic total shoulder replacement' OR 'conventional total shoulder arthroplasty' OR 'reverse total shoulder arthroplasty' OR 'reverse total shoulder replacement' OR 'tsa' OR 'rTSA' OR 'primary tsa*') AND ('upper extremity dominance' OR 'upper limb dominance' OR 'hand dominance' OR 'arm dominance' OR 'dominant hand' OR 'non-dominant hand' OR 'nondominant hand' OR 'dominant upper limb' OR 'non-dominant upper limb' OR 'nondominant upper limb' OR 'dominant limb' OR 'non-dominant limb' OR 'nondominant limb' OR 'dominant upper extremity' OR 'non-dominant upper extremity' OR 'nondominant upper extremity' OR 'dominant extremity' OR 'non-dominant extremity' OR 'nondominant extremity' OR 'dominant arm' OR 'non-dominant arm' OR 'nondominant arm' OR 'dominant-arm' OR 'nondominant-arm' OR 'non-dominant-arm' OR 'dominant group' OR 'non-dominant group' OR 'nondominant group' OR 'dominant side' OR 'non-dominant side' OR 'nondominant side') |
| Cochrane Library | ("Shoulder Arthroplasty" OR "Shoulder Replacement" OR "Anatomic Total Shoulder Arthroplasty" OR "Anatomic Total Shoulder Replacement" OR "Conventional Total Shoulder Arthroplasty" OR "Reverse Total Shoulder Arthroplasty" OR "Reverse Total Shoulder Replacement" OR TSA OR rTSA OR "primary TSA") AND ("Upper Extremity Dominance" OR "Upper Limb Dominance" OR "Hand Dominance" OR "Arm Dominance" OR "Dominant Hand" OR "Non-Dominant Hand" OR "Nondominant Hand" OR "Dominant Upper Limb" OR "Non-Dominant Upper Limb" OR "Nondominant Upper Limb" OR "Dominant Limb" OR "Non-Dominant Limb" OR "Nondominant Limb" OR "Dominant Upper Extremity" OR "Non-Dominant Upper Extremity" OR "Nondominant Upper Extremity" OR "Dominant Extremity" OR "Non-Dominant Extremity" OR "Nondominant Extremity" OR "Dominant Arm" OR "Non-Dominant Arm" OR "Nondominant Arm" OR "Dominant-Arm" OR "Nondominant-Arm" OR "Non-Dominant-Arm" OR "Dominant Group" OR "Non-Dominant Group" OR "Nondominant Group" OR "Dominant Side" OR "Non-Dominant Side" OR "Nondominant Side") |
